# Targeting Transcriptional Regulation with a CDK9 Inhibitor Suppresses Growth of Endocrine- and Palbociclib-Resistant ER^+^ Breast Cancers

**DOI:** 10.1158/0008-5472.CAN-23-0650

**Published:** 2023-10-06

**Authors:** Arany Soosainathan, Marjan Iravani, Rania El-Botty, John Alexander, Laura Sourd, Ludivine Morisset, Pierre Painsec, Rebecca Orha, Joanna Nikitorowicz-Buniak, Sunil Pancholi, Syed Haider, Mitch Dowsett, Elisabetta Marangoni, Lesley-Ann Martin, Clare M. Isacke

**Affiliations:** 1 The Breast Cancer Now Toby Robins Research Centre, The Institute of Cancer Research, London, United Kingdom.; 2 Translational Research Department, Institut Curie, Paris, France.; 3 Ralph Lauren Centre for Breast Cancer Research, Royal Marsden Hospital, London, United Kingdom.

## Abstract

The combination of endocrine therapy and CDK4/6 inhibitors such as palbociclib is an effective and well-tolerated treatment for estrogen receptor–positive (ER^+^) breast cancer, yet many patients relapse with therapy-resistant disease. Determining the mechanisms underlying endocrine therapy resistance is limited by the lack of ability to fully recapitulate inter- and intratumor heterogeneity *in vitro* and of availability of tumor samples from women with disease progression or relapse. In this study, multiple cell line models of resistant disease were used for both two-dimensional (2D)– and three-dimensional (3D)–based inhibitor screening. The screens confirmed the previously reported role of pro-proliferative pathways, such as PI3K–AKT–mTOR, in endocrine therapy resistance and additionally identified the transcription-associated cyclin-dependent kinase CDK9 as a common hit in ER^+^ cell lines and patient-derived organoids modeling endocrine therapy–resistant disease in both the palbociclib-sensitive and palbociclib-resistant settings. The CDK9 inhibitor, AZD4573, currently in clinical trials for hematologic malignancies, acted synergistically with palbociclib in these ER^+^*in vitro* 2D and 3D models. In addition, in two independent endocrine- and palbociclib-resistance patient-derived xenografts, treatment with AZD4573 in combination with palbociclib and fulvestrant resulted in tumor regression. Tumor transcriptional profiling identified a set of transcriptional and cell-cycle regulators differentially downregulated only in combination-treated tumors. Together, these findings identify a clinically tractable combination strategy for overcoming resistance to endocrine therapy and CDK4/6 inhibitors in breast cancer and provide insight into the potential mechanism of drug efficacy in targeting treatment-resistant disease.

**Significance:**

Targeting transcription-associated CDK9 synergizes with CDK4/6 inhibitor to drive tumor regression in multiple models of endocrine- and palbociclib-resistant ER^+^ breast cancer, which could address the challenge of overcoming resistance in patients.

## Introduction

Approximately 80% of breast cancers are estrogen receptor–positive (ER^+^) at diagnosis, with most patients receiving adjuvant endocrine therapy. Unfortunately, endocrine therapy resistance remains a significant clinical problem with 13%–41% ER^+^ breast cancers relapsing following adjuvant endocrine therapy (1). The major mechanisms identified in driving resistance are (i) acquisition of mutations in the estrogen receptor (*ESR1*) resulting in ligand-independent ER signaling, and (ii) rewiring of the cross-talk pathways between ER and growth factor receptor signaling resulting in ER activation in the absence of estrogen (E2) and/or enhanced growth factor signaling via Ras–Raf–MAPK or PI3K–AKT–mTOR converging on cyclin D to promote cell-cycle progression (2). Targeting the cell cycle with cyclin-dependent kinase (CDK)-4/6 inhibitors such as palbociclib, ribociclib, and abemaciclib in combination with aromatase inhibitors has been shown to improve clinical outcomes in patients with advanced ER^+^/HER2^−^ breast cancer (3), and has been approved as first- and second-line treatment options. Unfortunately, resistance to these targeted therapies has become the next hurdle in the treatment of advanced ER^+^ disease. As such, there is a clinical need for new treatments for patients with advanced endocrine-resistant, CDK4/6 inhibitor–resistant disease.

The challenge presented by ER^+^ breast cancers is the heterogeneity of the disease and mechanisms by which treatment resistance disease develops. Consequently, in this study multiple models of resistant ER^+^ disease were used for both two-dimensional (2D)– and three-dimensional (3D)–based inhibitor screening and *in vivo* validation.

## Materials and Methods

### Cell culture

The origin and culture conditions for the cell lines is listed in Supplementary Table S1. Cell lines were authenticated by short tandem repeats profiling (GenePrint 10 System; Promega), routinely screened for *Mycoplasma* (MycoAlert; Lonza) and used within 10–15 passages after thawing. Unless otherwise stated, estradiol and palbociclib were removed from the media of the parental and palbociclib-resistant cells, respectively, 48 hours prior to each experiment.

### Drugs

The 2D drug screen was performed with the SelleckChem 378 kinase inhibitor library plus 15 additional compounds, staurosporin (positive control) and DMSO (vehicle control; Supplementary Table S2). The 3D drug screen was performed with 71 compounds (Supplementary Table S3). For validation and *in vivo* studies, AZD4573, NVP-2, and palbociclib were purchased from MedChemExpress, and fulvestrant was purchased from Teva.

### 2D Drug screen

Drugs (10 nmol/L, 100 nmol/L, and 1 μmol/L final concentration) were prealiquoted into 384-well 2D culture plates (Greiner Bio-One) using an ECHO550 acoustic liquid handler prior to seeding 1,200–2,400 cells/well in 50 μL of cell line–specific media. Cell viability was assessed using CellTiter-Glo (Promega; day 5). Assay quality was assessed using calculation of Z-prime per plate, and only plates with Z-prime of ≥0.5 were taken for further analysis. Three independent biological replicates (each replicate formed of six plates) were performed to reduce the risk of false hit identification. To generate a percentage of control (POC) score, the raw luminescence value for each well was normalized by dividing this value by the median value of the negative controls on the plate. The mean of the POC scores of the three independent biological replicates was used to generate a mean response score for each drug, at each concentration tested.

### 3D Drug screen

Drugs were dispensed into ultra-low attachment round-bottomed 96-well 3D culture plates (Corning) using the Hamilton liquid handler prior to seeding 2,500 cells/well in 100 μL of culture medium. Cell viability was assessed using CellTiter-Glo (day 7). Assay quality was assessed using calculation of Z-prime per plate, and only plates with Z-prime of ≥0.5 were taken for further analysis. Three independent biological replicates for each cell line (each replicate formed of one plate) were performed to reduce the risk of false hit identification. A robust Z-score threshold was set at ≤−1.65 to classify and compare hits.

### Patient-derived organoid studies

Organoids were established from patient-derived xenografts (PDX) of ER^+^ primary or metastatic tumors (Supplementary Table S4; refs. 4, 5) as described elsewhere (6). Briefly, PDX tumors were dissociated with an enzymatic solution, embedded in 100 μL Matrigel (Corning, 354230), and plated in six-well tissue culture plates onto a 70 μL Matrigel base layer. After a 30-minute incubation, organoid medium consisting of advanced DMEM/F12 with 5% FBS, 10 mmol/L HEPES, 1× Glutamax, 1 μg/mL hydrocortisone, 50 μg/mL gentamicin, 10 ng/mL hEGF, 100 ng/mL FGF2, 10 μmol/L Y-27632, and 1 mmol/L N-acetyl cysteine was added. Medium was replenished every 3 to 4 days. Organoids were dissociated using TrypleExpress (Life Technologies, 12605010) for 20 minutes. Established organoids were treated with AZD4573 at different concentrations and cell viability assessed using CellTiter-Glo 3D [Promega, G9682 (v/v)] following 20-minute incubation at room temperature. Luminescence was measured using Tecan Infinite 200.

### *In vivo* PDX studies

All animal works were carried out in accordance with institutional guidelines and rules of the French ethics committee CEEA-IC (Comité d'Ethique en matière d'expérimentation animale de l'Institut Curie, national registration number: #118), project authorization number 02163.02. Generation of the ER^+^ PDX models HBCx-180 and HBCx-134-palboR31 have been described previously (5). Palbociclib (75 mg/kg) was administered orally 5 days per week, AZD4573 (15 mg/kg) was administered twice daily a 2-day-on/5-day-off schedule, fulvestrant (50 mg/kg) was administered by intramuscular injection once a week. Mice were weighed daily, and tumor size measured twice per week. Tumor volumes were calculated as *V* = *a* × *b*^2^/2, with *a* being the largest diameter, *b* the smallest. Tumor volumes were reported to the initial volume as relative tumor volume (RTV), and growth curves were established as a function of time. For each tumor, the percent change in volume was calculated as (*V*_f_ − *V*_0_/*V*_0_)/100, *V*_0_ being the volume at the beginning of treatment and *V*_f_ the final volume. Mice were culled 1 hour following the second AZD4573 treatment of the day.

### Transcriptional profiling of PDX tumors

Two micrograms of total RNA was used for RNA sequencing (RNA-seq) analysis (Azenta). See Supplementary Methods for details of RNA extraction and bioinformatic analysis.

### Statistical analysis

All statistical tests were performed in GraphPad Prism except RNA-seq and high-throughput screen data. Unless otherwise stated, dose–response curves and IC_50_ values were calculated using a four-parameter nonlinear regression.

### Data availability

RNA-seq data has been deposited in SRA submission ID: PRJNA933383. All other raw data are available upon request from the corresponding author.

## Results

The ER^+^ breast cancer cell lines used in this study (MCF7, SUM44, HCC1428, T47D, and ZR-75–1; Supplementary Table S1) were chosen to encompass a range of ER^+^ breast cancer molecular phenotypes. Each of the cell lines had been extensively cultured in the absence of exogeneous E2 to generate long-term estrogen-deprived (LTED) lines that proliferate in an E2-independent manner thus modeling breast cancer that has become resistant to aromatase inhibitor therapy (see Supplementary Methods; Supplementary Fig. S1). For MCF7 and SUM44, independent LTED lines harboring WT *ESR1* or *ESR1*-activating mutations were used.

### Kinase inhibitor screens of LTED breast cancer cell lines in 2D and 3D

**Figure 1.**
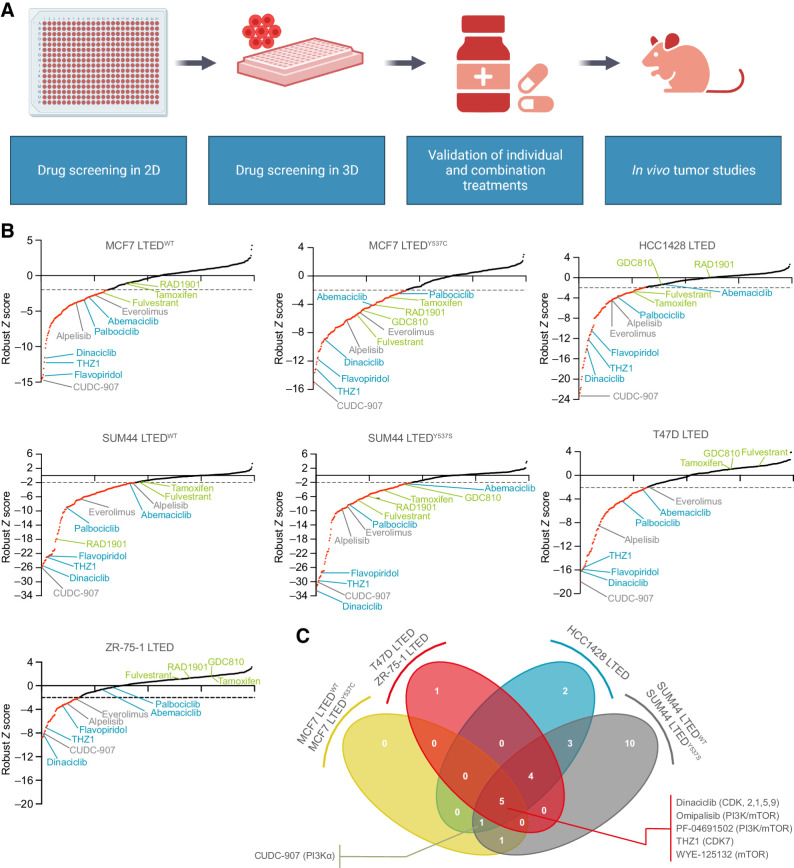
Two-dimensional kinase inhibitor screens in LTED breast cancer cell line models. **A,** Schematic representation of the study. **B,** 2D screens at 1 μmol/L final drug concentration (see Materials and Methods). Sigma plots represent robust Z-score rank order, with drugs achieving a robust Z-score ≤ −2 in red. Compounds causing ≥ 50% reduction in cell viability were classified as hits. Representative drugs of the following classes are highlighted. Green, targeting ER signaling; blue, targeting CDKs; gray, targeting PI3K–AKT–mTOR pathway. **C,** 2D screens at 100 nmol/L final drug concentration. Compounds causing ≥ 50% reduction in cell viability were classified as hits. Venn diagram illustrating the number of hits in common to three or four of the cell line groups is indicated. Full list is provided in Supplementary Fig. S2. (**A,** Created with BioRender.com.)

Seven LTED lines were subjected to high-throughput 2D kinase inhibitor screening (*n* = 393 drugs) at three concentrations (10 nmol/L, 100 nmol/L and 1 μmol/L). Treatment with 1 μmol/L drug concentrations (Fig. 1A and B; Supplementary Table S2), revealed three broad areas key to endocrine resistance: (i) the PI3K–AKT–mTOR pathways, with drugs targeting the pathway in all LTED cell lines; (ii) cell-cycle checkpoints, with CDK inhibitors frequently identified; and (iii) ER signaling, with ER inhibitors such as fulvestrant and tamoxifen featuring as recurrent hits. Next, a mean response score was calculated for each drug and each cell line, with those causing ≥50% reduction in cell viability categorized as hits. At the lower drug dose (100 nmol/L), the majority of LTED lines retained sensitivity to compounds targeting the PI3K–AKT–mTOR pathway and cell-cycle checkpoints (Fig. 1C; Supplementary Fig. S2A).

**Figure 2.**
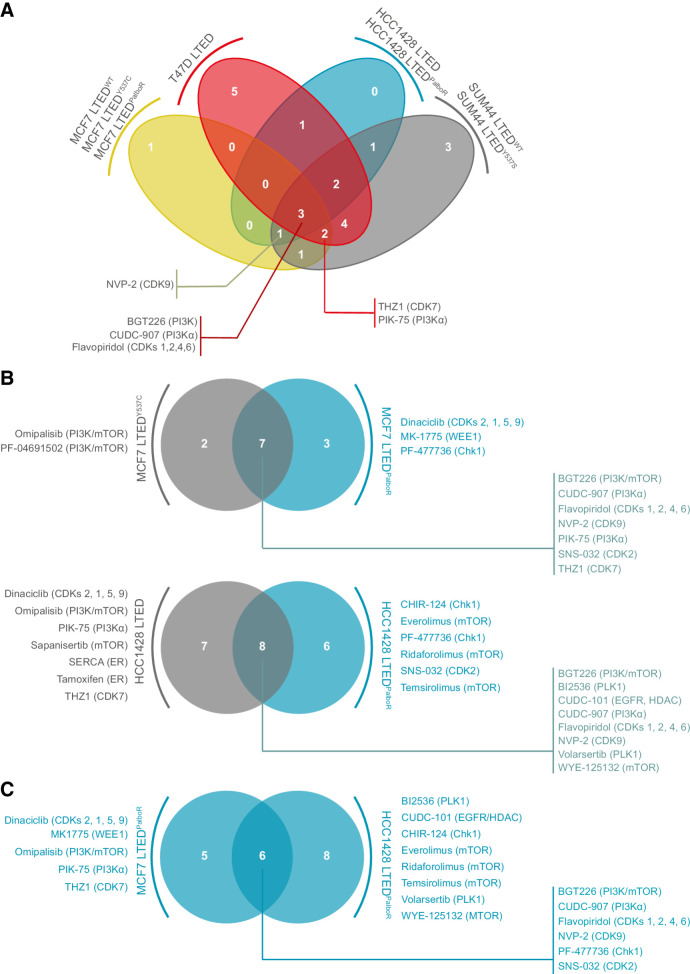
Three-dimensional kinase inhibitor screens in LTED and palbociclib-resistant breast cancer cell line models. Three-dimensional assays were performed at 250 nmol/L final drug concentration (see Materials and Methods). Compounds with a robust Z-score ≤1.65 were classified as hits. **A,** Venn diagram illustrating the number of hits common to three or four of the cell line groups indicated. **B,** Comparison of hits in palbociclib-sensitive MCF7 LTED and HCC1428 LTED breast cancer cell lines and their palbociclib-resistant (PalboR) derivatives. **C,** Comparison of the hits in the palbociclib-resistant cell lines MCF7 LTED^PalboR^ and HCC1428 LTED^PalboR^.

The design of the 3D drug screen using 71 drugs (Supplementary Table S3) was informed by the results of the 2D screen and included two palbociclib-resistant lines MCF7 LTED^PalboR^ and HCC1428 LTED^PalboR^ (Supplementary Table S1). 3D spheroid screening in triplicate at a single drug concentration (250 nmol/L) identified drugs targeting the PI3K–AKT–mTOR pathway in all cell lines, with the multi-CDK inhibitor flavopiridol also a common hit (Fig. 2A; Supplementary Fig. S2B). Drugs that were significant hits in three of the four cell line groups were: PIK-75 (PI3Kα inhibitor), the CDK7 inhibitor THZ1, and the CDK9 inhibitor NVP-2. The palbociclib-resistant models retained their sensitivity to PI3Kα inhibition; however, they were less sensitive to pan-PI3K inhibitors than the palbociclib-sensitive models (Fig. 2B). Comparison of the two palbociclib-resistant models indicates a shared common vulnerability to compounds targeting cell-cycle control, and CDK9 inhibitors (Fig. 2C). Of note, in addition to the palbociclib-resistant and -sensitive lines, CDK7 and/or CDK9 inhibitors targeted both WT (HCC1428) and mutant PIK3CA (MCF7) LTED cell lines.

The identification of PI3K–AKT–mTOR pathway as a key dependency in endocrine therapy and palbociclib-resistant breast cancers is well documented (7). In addition, agents targeting CDK7 have been shown to have favorable activity in several cancer types (8, 9), and early studies have shown promising results in administering the CDK7 inhibitor samuraciclib in combination with fulvestrant in a heavily pretreated cohort of patients with ER^+^ breast cancer (NCT03363893), with phase I trials also underway with an independent CDK7 inhibitor, SY-5609 (NCT0424716). Although the efficacy of CDK7 inhibition in endocrine-resistant, palbociclib-resistant breast cancer is currently being investigated (10), little is known as to the potential of targeting the transcriptional regulator CDK9 in ER^+^ breast cancers.

### Targeting CDK9 impairs viability in palbociclib-sensitive and -resistant LTED cells lines

**Figure 3.**
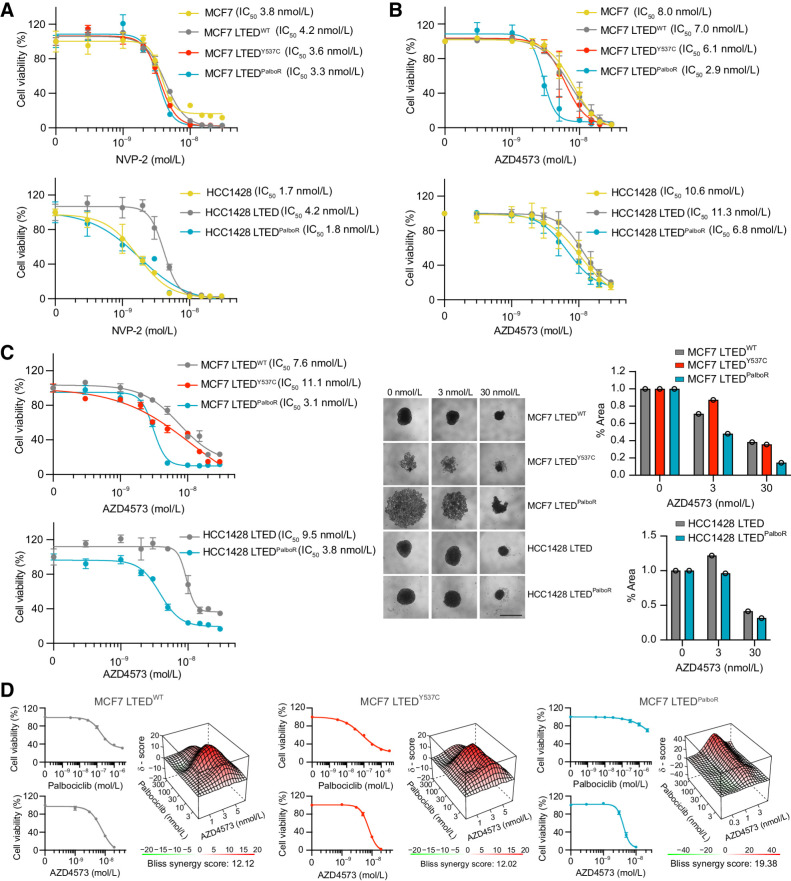
CDK9 inhibitors impair cell viability in 2D and 3D culture. **A** and **B,** A total of 4,000 to 8,000 cells were seeded in 96-well tissue culture plates and treated at day 1 and 3 with the CDK9 inhibitors NVP-2 (**A**) or AZD4573 (**B**) at the indicated concentrations. Cell viability was assessed using CellTiter-Glo (day 7). Mean values ± SEM. Data represent percentage NVP-2/AZD4573–treated viable cells compared with vehicle control. *n* = 4 technical replicates; *n* = 1 biological replicate. **C,** A total of 2,500 cells were seeded in 96-well ultra-low attachment round-bottomed plates. The resulting spheroids were treated with AZD4573 at days 3 and 6. Cell viability was assessed on day 10 (endpoint). Graphs showing percentage of viable AZD4573-treated cells compared with vehicle control. *n* = 1 biological replicate; *n* = 4 technical replicates. Mean values ± SEM. Representative images and quantification of spheroid area at endpoint following treatment with 0 nmol/L, 3 nmol/L, or 30 nmol/L AZD4573 are shown. Scale bar, 1 mm. **D,** A total of 3,000 to 5,000 MCF7 LTED^WT^, LTED^Y537C^, or LTED^PalboR^ cells were seeded in 2D 96-well tissue culture plates and treated with escalating doses of palbociclib and AZD4573 at day 1 and 3. Cell viability was assessed using CellTiter-Glo on day 7. Data representing percentage of viable cells compared with vehicle control. *n* = 1 biological replicate; *n* = 3 technical replicates; mean values ± SEM. Top, individual drug responses. Bottom, synergy plots. The Bliss synergy score references the most synergistic area of the plot.

For validation studies, the specific CDK9 inhibitor NVP-2 (Fig. 3A) and the chemically distinct inhibitor AZD4573 (Fig. 3B) known to induce tumor regression in a PDX model of AML (11) and now being examined in phase II trials for advanced hematological malignancies (NCT03263637, NCT04630756) were used. These compounds were chosen for their high selectivity for CDK9 over other kinases, including CDK7 (11, 12). Cell viability assays in 2D confirmed that both MCF7 and HCC1428 lines are sensitive to low doses of these drugs, findings corroborated by siRNA knockdown of CDK9 (Supplementary Fig. S3), but of great interest the palbociclib-resistant cell lines showed a lower IC_50_ than their MCF7 LTED^Y537C^ and HCC1428 LTED counterparts. Equivalent IC_50_ values were obtained in 3D spheroid assays (Fig. 3C), with all cell lines showing sensitivity to the CDK9 inhibitor AZD4573, and both the MCF7 and HCC1428 palbociclib-resistant lines showing increased sensitivity compared with their palbociclib-sensitive counterparts.

To assess whether CDK9 activity contributes to the mechanism of palbociclib resistance, the MCF7 LTED^Y537C^ line and its palbociclib-resistant derivative, as well as endocrine-resistant palbociclib-sensitive MCF7 LTED^WT^ cells, were treated with escalating doses of both palbociclib and AZD4573 (Fig. 3D). In 2D, all three MCF7 lines showed positive Bliss synergy scores >10, indicative of a synergistic effect, with the greatest effect seen in the MCF7 LTED^PalboR^ line (synergy score of 19.38), indicating that the development of palbociclib resistance may be accompanied by an increased reliance on CDK9 activity.

### Targeting CDK9 impairs viability in PDO and PDX models

**Figure 4.**
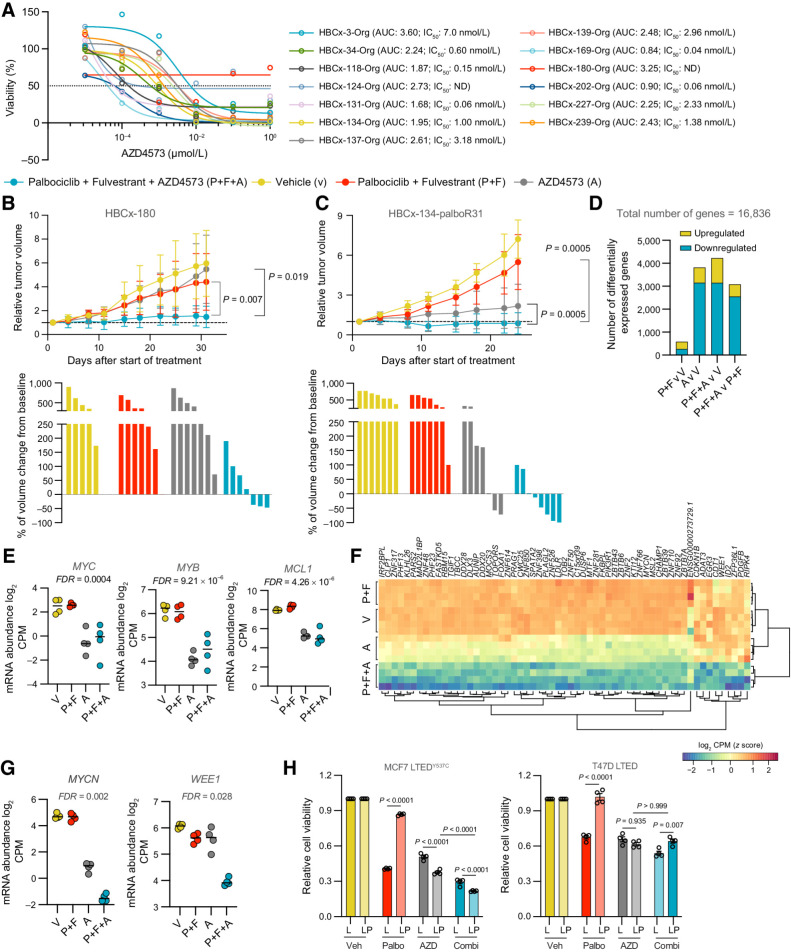
CDK9 inhibitor AZD4573 drives tumor regression in endocrine therapy and palbociclib-resistant PDXs. **A,** 2–5 × 10^4^ dissociated PDO cells were seeded per well in 96-well tissue culture plates in 10% Matrigel. The resulting organoids were treated with escalating doses of AZD4573 at day 2. Cell viability was assessed on day 7. Dose–response curves showing percentage of viable AZD4573-treated cells compared with vehicle control. *n* = 4 wells per PDO. Drug response represented by sigmoidal dose–response curve. IC_50_ and AUC (area under curve) values are shown. ND, not determined. **B** and **C,** HBCx-180 (**B**) and HBCx-134-palboR31 (**C**) PDXs were inoculated into 8-week-old Swiss nude mice. Xenografts were randomly assigned to different groups when tumors reached a volume of 100 to 200 mm^3^ and treated with vehicle, palbociclib + fulvestrant, AZD4573 or the combination of palbociclib + fulvestrant + AZD4573 (V, P+F, A, P+F+A). Top, relative tumor volumes ±SD. HBCx-180, *n* = 6–7; HBCx-134-palboR31, *n* = 7–10 mice per group (two-sided Mann–Whitney *U* test). Bottom, waterfall plots showing percent change in tumor volume from baseline. HBCx-180, *n* = 5–7; HBCx-134-palboR31, *n* = 7–8 mice per group. **D–G,** RNA-seq analysis of HBCx-134-palboR31–treated tumors**. D,** Number of genes differentially up- and downregulated in tumor treatment group comparisons, using significance thresholds of |log_2_FC| > 1 and FDR adjusted *P* < 0.05. **E,** Expression of *MYC*, *MYB*, and *MCL1* in tumor treatment groups. *P* values were estimated using edgeR's implementation of quasi-likelihood F test. **F,** Heat map of genes with synergistic downregulated expression in palbociclib + fulvestrant + AZD4573–treated tumors (log_2_FC > 1, FDR-adjusted *P* < 0.05). **G,** Expression of *MYCN* and *WEE1* in tumor treatment groups. *P* values were estimated using edgeR's implementation of quasi-likelihood F-test. **H,** 5,000 MCF7 LTED^Y537C^ or T47D LTED (L) or LTED^PalboR^ (LP) cells were seeded in 96-well ultra-low attachment round-bottomed plates and resulting spheroids treated with AZD4573 (10 nmol/L) or palbociclib (1 μmol/L) or combination at days 3 and 6. Cell viability was assessed on day 10. Data represents relative cell viability compared with vehicle control (*n* = 4 biological replicate, *n* = 5 technical replicates per biological replicate; mean values ±SEM; two-way ANOVA with Sidak multiple comparisons test, and confidence intervals of 95%). AZD, AZD4573; Combi, AZD4573 + palbociclib; Palbo, palbociclib; Veh, vehicle.

To validate our results in more clinically relevant models, we determined the efficacy of AZD4573 inhibition on 13 patient-derived organoids (PDO) established from PDX tumors of ER^+^ patient primary or metastatic tumors. These PDOs harbored a range of genetic and copy number alterations and, for 5 of the PDOs, were derived from patients who had progressed on CDK4/6 and aromatase inhibitor treatment (Fig. 4A; Supplementary Table S4). Eleven of 13 PDOs had IC_50_ values ≤ 7 nmol/L AZD4573 and only two PDOs showed no response to treatment, demonstrating that a broad range of ER^+^ PDOs are sensitive to AZD4573 as a monotherapy. Finally, we extended this study to PDX *in vivo* models of palbociclib intrinsic and acquired resistance. First AZD4573 treatment was assessed in the HBCx-180 PDX model developed from a patient with ER^+^ metastatic breast cancer, who had progressed on treatment with the aromatase inhibitor letrozole and palbociclib (5). Consistent with the HBCx-180 PDO *in vitro* results (Fig. 4A) and the intrinsic palbociclib resistance observed in the patient, neither AZD4573 alone nor dual therapy with palbociclib and fulvestrant impacted PDX tumor growth; however, the triple combination of AZD4573, palbociclib, and fulvestrant resulted in stable disease (Fig. 4B). The second PDX model, HBCx-134-palboR31, was established from a palbociclib-sensitive tumor and developed into a palbociclib-resistant model by *in vivo* palbociclib treatment (5). In this model of acquired palbociclib resistance, there was no impairment in tumor growth in mice treated with palbociclib and fulvestrant. Consistent with the PDO *in vitro* data for the HBCx-134 parental line (Fig. 4A), AZD4573 treatment alone slowed tumor growth and resulted in tumor regression in two mice; however, a profound effect on tumor growth in all mice was observed with the triple fulvestrant, palbociclib, and AZD4573 combination (Fig. 4C).

CDK9 is a transcriptional regulator acting downstream of CDK7 to promote phosphorylation of RNA Pol II and transcriptional elongation. RNA-seq analysis of HBCx-134-palboR31 tumors from mice treated with AZD4573 revealed extensive transcriptional downregulation, including that of known CDK9 target genes *MYC*, *MYB*, and *MCL1* (13–16; Fig. 4D and E; Supplementary Fig. S4A and S4B; Supplementary Table S5). Pathway analysis of AZD4573-treated tumors confirmed the effective downregulation of RNA polymerase II–mediated transcription, whereas in palbociclib plus fulvestrant-treated tumors, in the absence of AZD4573, downregulated pathways were enriched for cell-cycle control processes (Supplementary Fig. S4C).

Given the synergistic effect observed when utilizing AZD4573 in combination with palbociclib and fulvestrant (Fig 4C), we identified genes that showed synergistic downregulation in the combination treatment arm while accounting for the univariate effect of other treatment groups (Fig. 4F; Supplementary Table S6). The major cluster of differentially expressed genes was dominated by transcriptional regulators, such as the known CDK9 target gene *MYCN* (17), whose expression was further diminished by combination treatment (Fig. 4G). The minor cluster comprised genes, including cell-cycle regulators, significantly downregulated in the triple combination arm but with broadly similar expression in the other three arms, as exemplified by the expression of *WEE1* (Fig. 4G) previously shown to mediate resistance to CDK4/6 inhibitors (18). Of note, expression of other key regulators of CDK9 activity and palbociclib resistance such as *BRD4* and *CCNE1* showed a similar expression trend (Supplementary Fig. S4D). To validate these findings *in vitro* we compared the response of MCF7 LTED and T47D LTED lines to combination treatment. As expected, both LTED^PalboR^ lines showed resistance to palbociclib and sensitivity to AZD4573 but only the MCF7 LTED^PalboR^ line, which express higher levels of *MYC*, *MYB*, *CCNE1*, *CCNE2*, *BRD4*, *MYCN*, and *WEE1* (Supplementary Fig. S5), showed an enhanced response to the combination of AZD4573 and palbociclib (Fig. 4H).

## Discussion

Approximately 20 CDKs can be grouped into the cell cycle–associated CDKs, such as the palbociclib targets CDK4 and CDK6, and transcription-associated CDKs, which include CDK7 and CDK9. CDK9, in complex with cyclin T, forms the positive transcription elongation factor b (P-TEFb), which is recruited by BRD4 to paused transcription sites to facilitate RNA polymerase II–mediated transcriptional elongation (13, 14). In both hematologic malignancies and solid tumors, there is increasing evidence that CDK9 activity is required to maintain sufficient levels of short half-life transcripts and proteins such as mixed-lineage leukemia (MLL)-fusion genes products, the antiapoptotic myeloid cell leukaemia-1 (MCL1) protein, and the proto-oncogenes MYB and MYC, which cancer cells are dependent upon for their survival (13–16). This has led to the development of CDK9 small-molecule inhibitors and PROTACs (19).

To date, clinical trials of CDK9 inhibitors have been restricted to hematologic malignancies (15, 20) where, despite positive preclinical results, the efficacy as monotherapy in clinical trials has been mostly mediocre. More encouraging outcomes have been in combination studies where there is evidence of CDK inhibitors potentiating the efficacy of other agents such as bortezomib, doxorubicin, and venetoclax (20). In ER^+^ breast cancer cell lines, CDK9 targeting has been shown to reduce the level of *MYC* and *MYB* expression and E2-independent tumor cell proliferation (21, 22) but little information is available of the effectiveness of CDK9 inhibition in reducing tumor growth *in vivo*. However, a new selective CDK9 inhibitor, KB-0742 has been shown to display potent antitumor activity in prostate cancer models and to downregulate key androgen receptor–driven transcriptional pathways (23) while KB-0742 and the D11 CDK9 inhibitor have been reported to target MYC-high triple-negative breast cancers (24, 25).

Current standard of care for advanced ER^+^ breast cancer is endocrine therapy in combination with CDK4/6 inhibitors and although these treatments have had significant impact on extending progression-free and overall survival, the development of therapy-resistant disease remains an inevitability for many patients. The findings presented here provide evidence for the utility of inhibitors against the transcription-associated CDK, CDK9, in endocrine therapy–resistant disease in both the CDK4/6 inhibitor–sensitive and -resistant settings. Moreover, in both PDX and cell line models we demonstrate that AZD4573 can act synergistically with palbociclib with greatest synergy seen at the IC_50_ value of AZD4573 (∼3 nmol/L) and between 300 and 3000 nmol/L palbociclib. In patients with advanced ER^+^ breast cancer receiving daily oral doses of 125 mg palbociclib, the mean plasma concentration of palbociclib at steady-state is 116 ng/mL (259 nmol/L; ref. 26), indicating that CDK9 inhibitor synergy in palbociclib-resistant cells is achieved at, or near, clinically relevant concentrations of palbociclib, and could translate into using subtherapeutic combination dosing to avoid toxicities.

## Supplementary Material

Supplementary Data Supplementary Methods

Supplementary Figure S1 Supplementary Figure S1

Supplementary Figure S2 Supplementary Figure S2

Supplementary Figure S3 Supplementary Figure S3

Supplementary Figure S4 Supplementary Figure S4

Supplementary Figure S5 Supplementary Figure S5

Supplementary Tables Supplementary Tables S1, S2, S3, S4, S5a, S5b, S5c, S5d, S6
