# Supplementary material for: Targeting Transcriptional Regulation with a CDK9 Inhibitor Suppresses Growth of Endocrine- and Palbociclib-Resistant ER+ Breast Cancers
Source: Cancer Res. 2023 Oct 6;84(1):17–25. doi: 10.1158/0008-5472.CAN-23-0650 (PMC10758688; doi:10.1158/0008-5472.CAN-23-0650)
Supplement: Supplementary Data — Supplementary Methods [file can-23-0650_supplementary_data_suppsm.pdf]

Targeting transcriptional regulation with a CDK9 inhibitor suppresses growth of endocrine- and palbociclib-resistant ER^+ breast cancers

Soosainathan et al.

Supplementary Methods

Supplementary Tables (provided as a tabbed Excel file)

Supplementary Table S1 | Breast cancer cell lines and culture medium

Supplementary Table S2 | List of drugs used in the 2D screen and their targets

Supplementary Table S3 | List of drugs used in the 3D screen and their targets

Supplementary Table S4 | Characteristics of PDOs

Supplementary Table S5 | Differential gene expression (RNA-Seq) between treatment groups

(a) vehicle versus palbociclib + fulvestrant

(b) vehicle versus AZD4573

(c) vehicle versus palbociclib + fulvestrant + AZD4573

(d) palbociclib + fulvestrant versus palbociclib + fulvestrant + AZD4573

Supplementary Table S6 | RNA-Seq analysis to test the synergistic effect of palbociclib + fulvestrant + AZD4573 combination treatment

Supplementary Figures

Supplementary Figure S1 | Validation of LTED cell lines

Supplementary Figure S2 | Kinase inhibitor screens in LTED and palbociclib-resistant breast cancer cell line models

Supplementary Figure S3 | Effect of CDK9 siRNA on cell viability

Supplementary Figure S4 | Gene expression profiling of PDX tumors

Supplementary Figure S5 | RTqPCR analysis of cell lines

Supplementary Methods

Cell lines

Characterization of the SUM44 LTED cell lines harboring an *ESR1*^{Y537S} activating point mutation have been previously described (1). This cell line is designated SUM44 LTED^{Y537S}. Validation by ddPCR showed that this mutation was detectable from twelve weeks of transfer to an E2-deprived medium, and thereafter the variant allele frequency increased up to 50%, indicating temporal enrichment of *ESR1* mutations through estrogen deprivation. As part of the same study, independently derived MCF7 LTED lines were characterized in a similar fashion, showing that one MCF7 LTED line harbored an *ESR1*^{Y537C} activating mutation (termed MCF7 LTED^{Y537C}) while the other was wild-type for *ESR1* (termed MCF7 LTED^{WT}). A further LTED model of SUM44 cells that were wild-type for *ESR1* (termed SUM44 LTED^{WT}) was kindly provided by Dr Oesterreich's group (University of Pittsburgh) (2).

Endocrine-resistant, palbociclib-resistant models were generated by long-term culture of LTED cell lines in the continuous presence of 1 μM palbociclib until resistance developed (approximately six months) (3). The palbociclib-resistant MCF7 LTED model (termed MCF7 LTED^{PalboR}) was generated from the MCF7 LTED^{Y537C} cell line (i.e. harboring the activating *ESR1* point mutation Y537C). The palbociclib-resistant HCC1428 and T47D LTED models (termed HCC1428 LTED^{PalboR} and T47D LTED^{PalboR} were generated from the HCC1428 LTED and T47D LTED cell lines.

Drug screen analysis

Screen assay quality was assessed using calculation of Z-prime (4). The R package cellHTS2 was used to calculate the robust Z-score (5). For combination assays, CellTiter-Glo data was inputted into SynergyFinder (6) to calculate the Bliss synergy score.

siRNA transfection

A reverse transfection protocol using SMARTpools of siRNA was performed for all siRNA transfection experiments. Lyophilized oligonucleotides were reconstituted in siRNA buffer to 20 μM and aliquots were stored at -20°C. ON-TARGETplus SMARTpools consist of four different siRNAs targeting the same gene, and the non-targeting control pool was made up of four non-targeting siRNAs. A final concentration of 25 nM siRNA was used for all experiments unless otherwise stated. siRNA was incubated with the lipid transfection Lipofectamine RNAiMAX for 15 minutes. Cell suspension was then added at the required density and incubated for 24 hours. For real-time quantitative polymerase chain reaction (RTqPCR), cells were then washed with ice-cold PBS and stored at -80°C until RNA was extracted. For Western blotting, exchange of fresh culture media was performed at 24 hours, with protein extraction occurring on day 5.

RNA extraction and RTqPCR

Cells for RNA extraction were seeded in 6 cm plates. Growth medium was aspirated from the plates, and cells were washed with ice-cold PBS. 350 μL of ice-cold RLT lysis buffer containing 1:100 β-mercaptoethanol was added to each plate. Cells were detached by scraping. mRNA was extracted from these cell lysates using the Qiagen RNeasy kit, performed according to the manufacturer’s protocol. RNA was eluted in 40 μL nuclease-free water, and concentration determined by measuring a 2 μL sample on the Nanodrop-8000 spectrophotometer. cDNA was produced by reverse transcribing 500 ng of RNA using the Qiagen Quantitect kit, according to the manufacturer’s protocol. RNA from *ex vivo* tumors were extracted using RNAeasy plus mini kit, briefly tumor chunks were placed in 2mL of ice-cold RLT lysis buffer containing 1:100 β-mercaptoethanol in Precellys tubes for homogenization on ice, 350μL of the homogenized solution was transferred to Qiashredder tube following manufacture’s instruction. Quantitative PCR reactions were performed using 11.25 ng cDNA, 5 μL 2x qPCR mastermix, and 0.5 μL Taqman Gene Expression Assay probe in 10 μL reaction. The QuantStudio6-Flex sequence detection system was used to perform relative quantification,

with all reactions performed in triplicate. Data analysis was performed using the Applied Biosystems QuantStudio 6 software. The endogenous control *B2M* was used to perform normalization, and all expression data was normalized to the non-targeting control.

ddPCR

Droplet digital PCR (ddPCR) was performed on a QX-200 ddPCR system using TaqMan chemistry with assays developed for *ESR1* (12004118: L536R, c.1607T>G; Y537C, c.1610A>G; D538G, c.1613A>G; E380Q, c.1138G>C; 12003910: Y537S, c.1610A>C; Y537N, c.1609T>A; S463P, c.1387T>C; Bio-Rad) multiplex hotspot mutations. FAM-labelled probes were designed for the mutant allele while HEX-labelled probes were designed for the corresponding wild-type allele. Primers and probes were used at a final concentration of 900 nM and 250 nM respectively. PCR reactions (20 µL) containing 10 ng of cell line DNA (approximately 1,500 diploid genomes equivalents), 10 µL ddPCR Supermix for probes (Bio-Rad) and 1 µL multiplex Bio-Rad assays were prepared and partitioned into a median of 20,000 droplets per sample in a manual droplet generator according to manufacturer's instructions. Emulsified PCR reactions were run on 96-well plates on a G-Storm GS4 thermal cycler with following conditions 95°C for 10 min followed by 40 cycles of 95°C for 15 sec and 52°C for 60 sec, followed by 10 min incubation at 98°C. The temperature ramp increment was 2.5°C/sec for all steps. Plates were read on a Bio-Rad QX-200 droplet reader using QuantaSoft v1.7.4 software from Bio-Rad to assess the number of droplets positive for mutant DNA, wild-type DNA, both, or neither. A non-targeting control well with no DNA was included for each assay in each run. A minimum of 10,000 droplets total and 2 FAM positive droplets were required for an assay to be considered successful. Cell lines that showed a mutant population by a multiplex ddPCR assay were validated with the identified mutation as a singleplex ddPCR as described above.

Immunoblotting

Cells were lysed in RIPA lysis buffer. Protein concentrations were determined using a Direct Detect Spectrometer (Millipore) or a Bradford assay. 15 μg protein samples were run on gels, transferred onto PVDF membranes, the membranes washed 3x for 5 minutes in TBS-T before incubation in the relevant primary antibody at 4°C overnight on an orbital shaker, washed 3x for 5 minutes in TBS-T before incubation in secondary antibody diluted in blocking buffer for 1 hour at room temperature. Membranes were then washed 3x for 5 minutes in TBS-T and developed in ECL substrate prior to imaging on the Bio-Rad imager.

RNA-Seq analysis

RNA-Seq profiling on xenograft tumors generated 55.3 to 74.8 million paired-end reads per sample. To evaluate the library quality, FastQC and FastQ Screen (7) were run on all FASTQ files and a summary report was generated using MultiQC (v1.9) (8). FASTQ reads were trimmed using Trim Galore (v0.6.6). Paired-end reads (150bp) were aligned to the human reference genome GRCh38, using STAR 2.7.6a (9) with --quantMode GeneCounts and --twopassMode Basic alignment settings. Annotation file used for feature quantification was downloaded from GENCODE (v22) in GTF file format. Differential mRNA abundance analysis between treatment groups (**Supplementary Table S5**) was performed using R package edgeR (v3.28.1) (10) in R statistical programming environment (v3.6.0). Genes with low expression were filtered out using edgeR's function 'filterByExpr()' with default parameters. Raw counts were normalized using edgeR's TMM (trimmed mean of M-values) method and differential mRNA abundance was performed using the quasi-likelihood (QL) F-test using the model *~0 + group*. To test synergistic effect of combination treatment (palbociclib + fulvestrant + AZD4573), coefficient of the interaction term was examined using the edgeR contrast: *(Palbociclib_Fulvestrant-vehicle)-(Palbociclib_Fulvestrant_AZD4573-AZD4573).* Results were further annotated using ENSEMBL gene annotations with the R package org.Hs.eg.db (v3.10.0) (see **Supplementary Table S6**). For GO enrichment analysis, ShinyGO 0.77 (http://bioinformatics.sdstate.edu/go/) was used (11).

References

1. Martin LA, Ribas R, Simigdala N, Schuster E, Pancholi S, Tenev T*, et al.* Discovery of naturally occurring ESR1 mutations in breast cancer cell lines modelling endocrine resistance. *Nat Commun* 2017;**8**(1):1865 doi 10.1038/s41467-017-01864-y.
2. Sikora MJ, Jacobsen BM, Levine K, Chen J, Davidson NE, Lee AV*, et al.* WNT4 mediates estrogen receptor signaling and endocrine resistance in invasive lobular carcinoma cell lines. *Breast Cancer Res* 2016;**18**(1):92 doi 10.1186/s13058-016-0748-7.
3. Pancholi S, Ribas R, Simigdala N, Schuster E, Nikitorowicz-Buniak J, Ressa A*, et al.* Tumour kinome re-wiring governs resistance to palbociclib in oestrogen receptor positive breast cancers, highlighting new therapeutic modalities. *Oncogene* 2020;**39**(25):4781-97 doi 10.1038/s41388-020-1284-6.
4. Zhang JH, Chung TD, Oldenburg KR. A Simple Statistical Parameter for Use in Evaluation and Validation of High Throughput Screening Assays. *J Biomol Screen* 1999;**4**(2):67-73 doi 10.1177/108705719900400206.
5. Malo N, Hanley JA, Cerquozzi S, Pelletier J, Nadon R. Statistical practice in high-throughput screening data analysis. *Nat Biotechnol* 2006;**24**(2):167-75 doi 10.1038/nbt1186.
6. Ianevski A, Timonen S, Kononov A, Aittokallio T, Giri AK. SynToxProfiler: An interactive analysis of drug combination synergy, toxicity and efficacy. *PLoS Comput Biol* 2020;**16**(2):e1007604 doi 10.1371/journal.pcbi.1007604.
7. Wingett SW, Andrews S. FastQ Screen: A tool for multi-genome mapping and quality control. *F1000Res* 2018;**7**:1338 doi 10.12688/f1000research.15931.2.
8. Ewels P, Magnusson M, Lundin S, Kaller M. MultiQC: summarize analysis results for multiple tools and samples in a single report. *Bioinformatics* 2016;**32**(19):3047-8 doi 10.1093/bioinformatics/btw354.
9. Dobin A, Davis CA, Schlesinger F, Drenkow J, Zaleski C, Jha S*, et al.* STAR: ultrafast universal RNA-seq aligner. *Bioinformatics* 2013;**29**(1):15-21 doi 10.1093/bioinformatics/bts635.
10. Robinson MD, McCarthy DJ, Smyth GK. edgeR: a Bioconductor package for differential expression analysis of digital gene expression data. *Bioinformatics* 2010;**26**(1):139-40 doi 10.1093/bioinformatics/btp616.
11. Ge SX, Jung D, Yao R. ShinyGO: a graphical gene-set enrichment tool for animals and plants. *Bioinformatics* 2020;**36**(8):2628-9 doi 10.1093/bioinformatics/btz931.
