# Supplementary material for: Targeting Transcriptional Regulation with a CDK9 Inhibitor Suppresses Growth of Endocrine- and Palbociclib-Resistant ER+ Breast Cancers
Source: Cancer Res. 2023 Oct 6;84(1):17–25. doi: 10.1158/0008-5472.CAN-23-0650 (PMC10758688; doi:10.1158/0008-5472.CAN-23-0650)
Supplement: Supplementary Figure S1 [file can-23-0650_supplementary_figure_s1_suppsf1.pdf]

Supplementary Figure 1

A

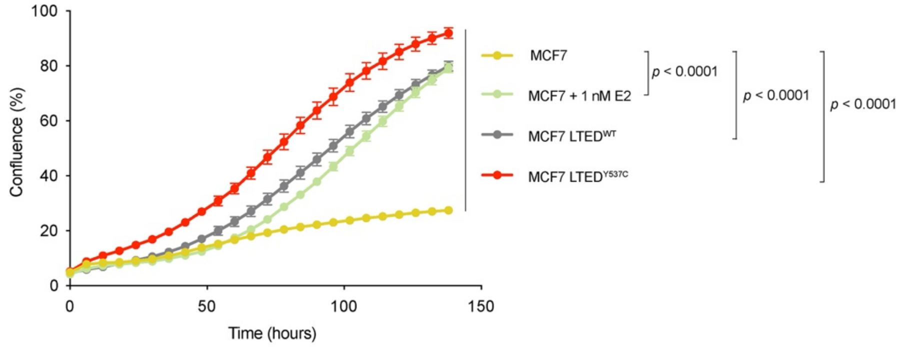

B

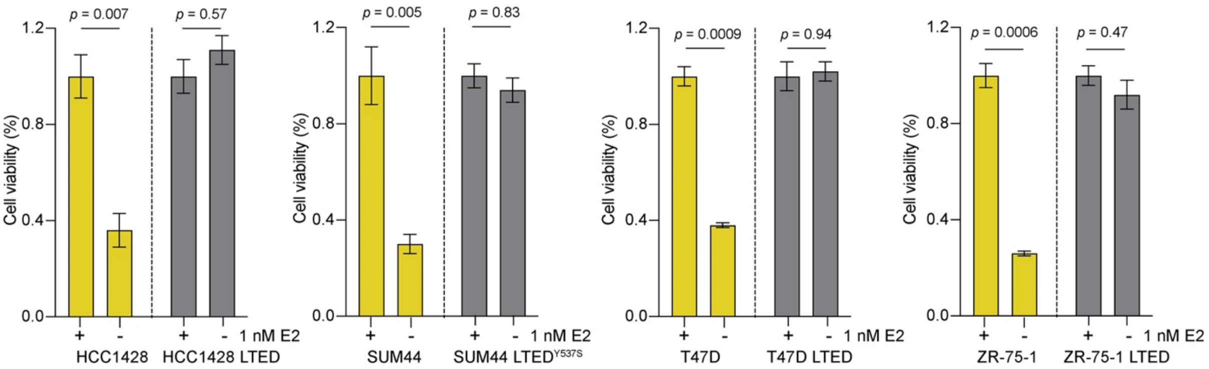

Supplementary Figure S1. Validation of LTED cell lines. **A,** Proliferation of parental and LTED MCF7 lines in E2-deprived conditions. 1.5 x 10^4 cells/well of MCF7, MCF7 $LTED^{WT}$ and MCF7 $LTED^{Y537C}$ cell lines were seeded into 24-well plates tissue culture plates in RPMI 1640 media supplemented with 10% DCC-FBS with/without supplemental E2. Cell proliferation was monitored over 138 hours using the Incucyte, with images taken every 6 hours. Graph shows mean changes in confluence relative to time zero ±SEM. *n*=2 biological replicates, *n*=6 technical replicates (two-way ANOVA with Sidak's multiple comparisons test, and confidence intervals of 95%). **B,** 2000-4000 cells/well of parental HCC1428, SUM44, T47D, and ZR-75-1 cells and their LTED derivatives were seeded into 96-well tissue culture plates in RPMI 1640 media supplemented with 10% DCC-FBS with/without E2 (as indicated). After 5 days, cell viability was assessed using CellTiter-Glo. Graphs show mean percentage change in viability relative to E2-treated parental cell line. *n*=2 biological replicates, *n*=8 technical replicates, ±SEM (one-way ANOVA with Sidak's multiple comparisons test, and confidence intervals of 95%).
