# Supplementary material for: Targeting Transcriptional Regulation with a CDK9 Inhibitor Suppresses Growth of Endocrine- and Palbociclib-Resistant ER+ Breast Cancers
Source: Cancer Res. 2023 Oct 6;84(1):17–25. doi: 10.1158/0008-5472.CAN-23-0650 (PMC10758688; doi:10.1158/0008-5472.CAN-23-0650)
Supplement: Supplementary Figure S2 [file can-23-0650_supplementary_figure_s2_suppsf2.pdf]

Supplementary Figure 2

A

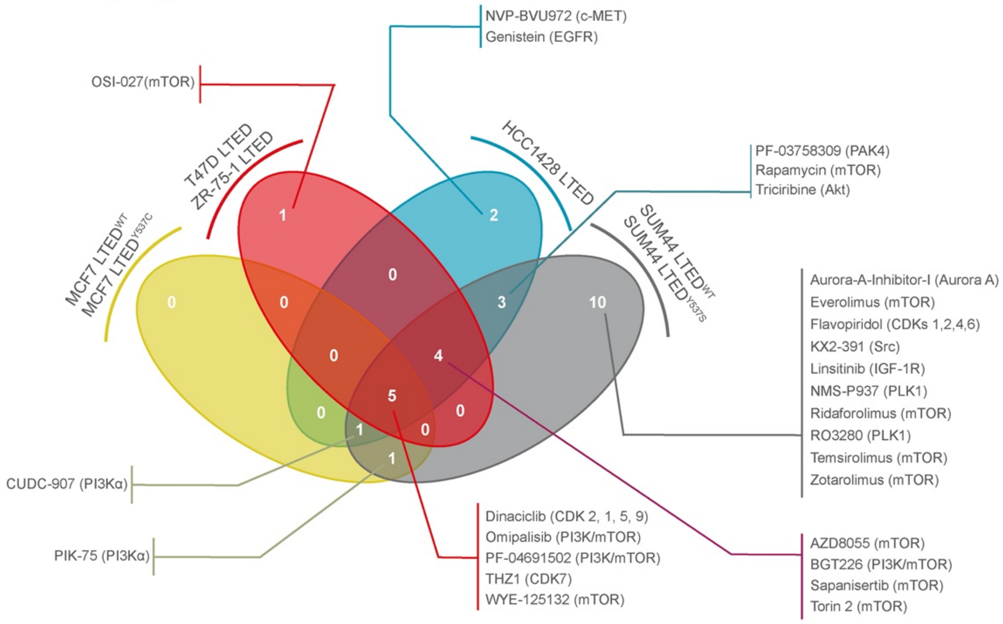

B

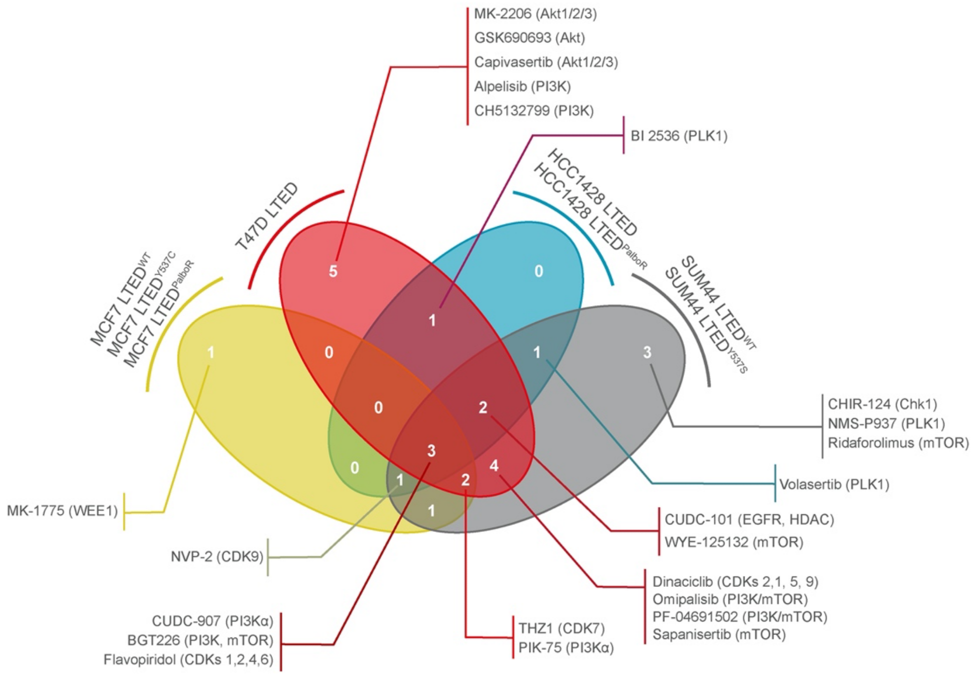

Supplementary Figure S2. Kinase inhibitor screens in LTED and palbociclib-resistant breast cancer cell line models. **A,** Extended data associated with **Fig. 1C**. 2D screens at 100 nM final drug concentration. Compounds causing ≥ 50% reduction in cell viability were classified as hits. **B,** Extended data associated with **Fig. 2A**. 3D assays were performed at 250 nM final drug concentration. Compounds with robust Z-score <-1.65 were classified as hits.
