# Supplementary material for: Targeting Transcriptional Regulation with a CDK9 Inhibitor Suppresses Growth of Endocrine- and Palbociclib-Resistant ER+ Breast Cancers
Source: Cancer Res. 2023 Oct 6;84(1):17–25. doi: 10.1158/0008-5472.CAN-23-0650 (PMC10758688; doi:10.1158/0008-5472.CAN-23-0650)
Supplement: Supplementary Figure S3 [file can-23-0650_supplementary_figure_s3_suppsf3.pdf]

Supplementary Figure 3

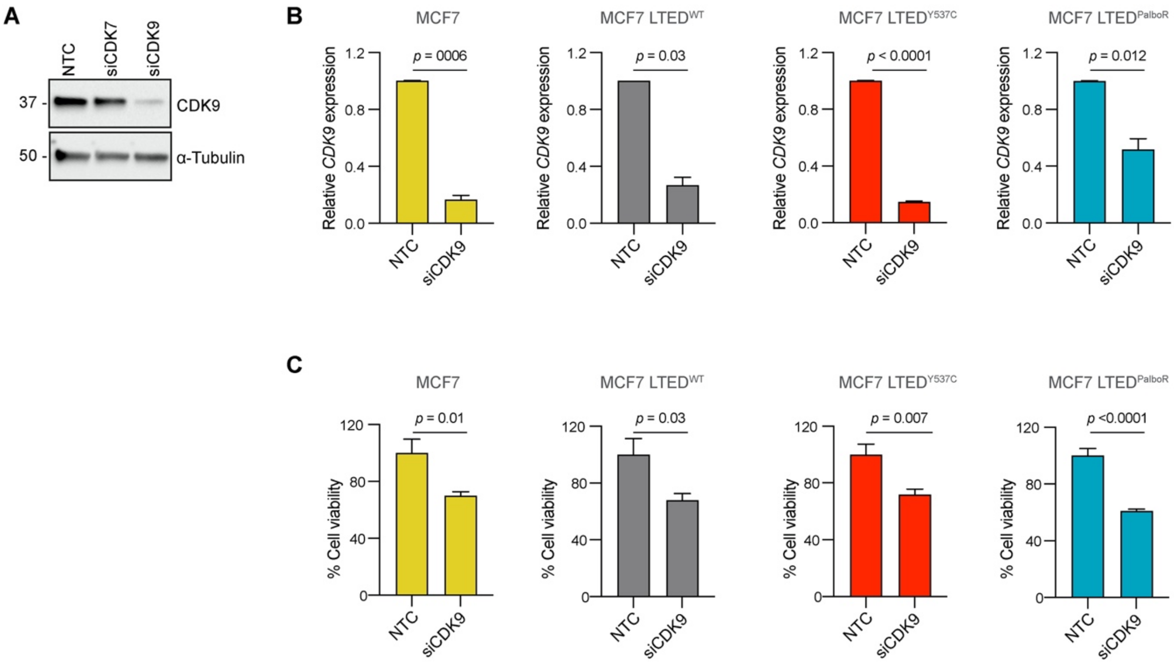

Supplementary Figure S3. Effect of CDK9 siRNA on cell viability. **A,B,** 1 x 10^6 - 5 x 10^5 cells were reverse transfected with 25 nM siNTC or 10 nM siCDK9 in 6 cm plates, cells were lysed after 4 days. **A,** Western blot show abundance of CDK9 in the MCF7 $LTED^{PalboR}$ cells. αTubulin (2125, Cell Signaling, 1:1000), CDK9 (2316, Cell Signaling, 1:1000). Molecular size markers are in kDa. **B**, RTqPCR analysis of *CDK9* expression. *n*=2 biological replicates, *n*=3 technical replicates ±SEM (two-tailed, unpaired *t*-test) Assay IDs: CDK9, Hs00977896_g1; *B2M*, Hs00187842_m1. **C,** 4000-8000 cells were seeded in 96-well plates and reverse transfected as panel A. Barplots represent percentage cell viability in cell lines transfected with siRNA targeting CDK9 (siCDK9) relative to non-targeting control (NTC) (*n*=2 biological replicates, *n*=6 technical replicates, ±SEM per cell line, two-tailed, unpaired *t*-test).
