# Supplementary material for: Targeting Transcriptional Regulation with a CDK9 Inhibitor Suppresses Growth of Endocrine- and Palbociclib-Resistant ER+ Breast Cancers
Source: Cancer Res. 2023 Oct 6;84(1):17–25. doi: 10.1158/0008-5472.CAN-23-0650 (PMC10758688; doi:10.1158/0008-5472.CAN-23-0650)
Supplement: Supplementary Figure S4 [file can-23-0650_supplementary_figure_s4_suppsf4.pdf]

Supplementary Figure 4

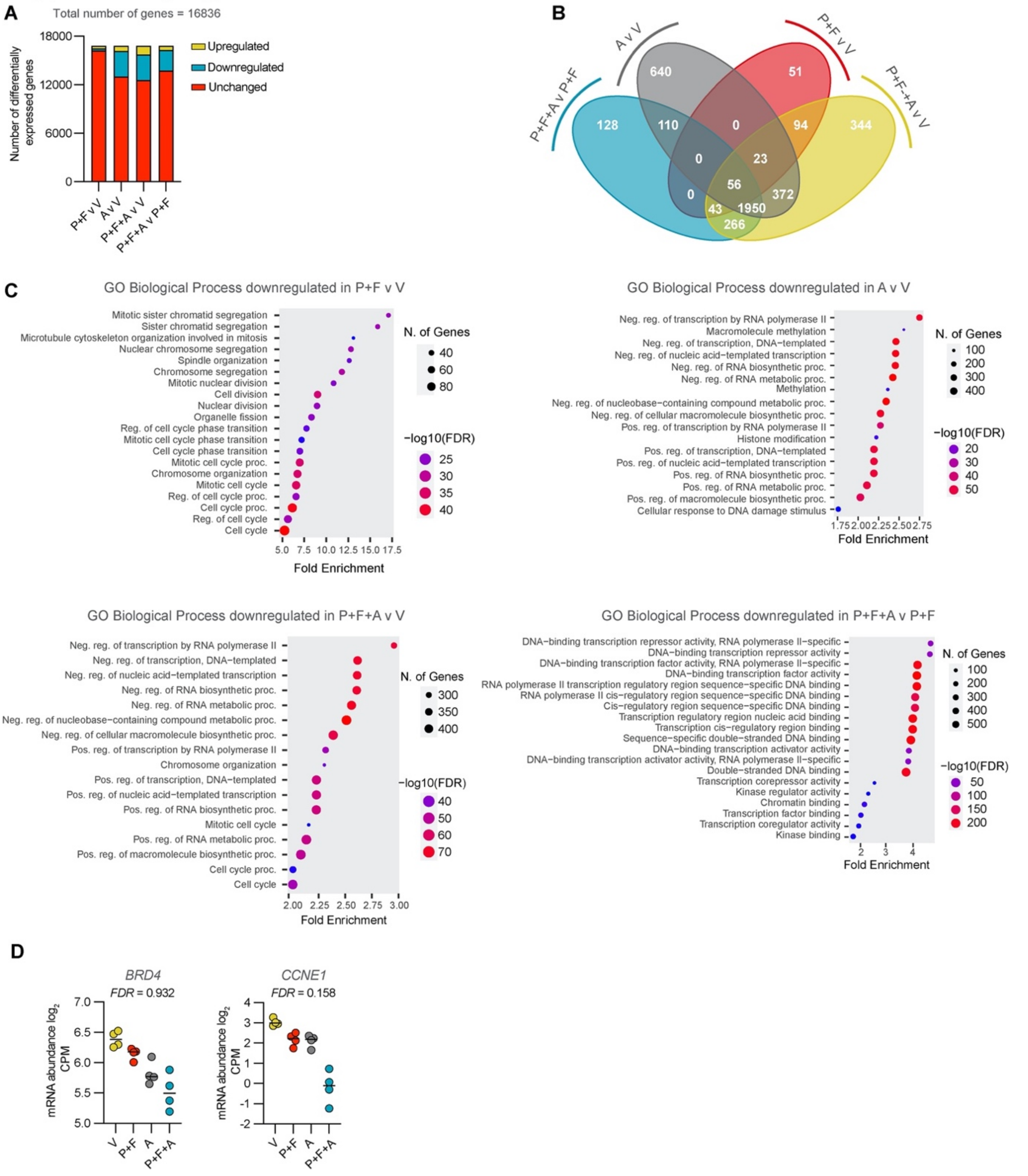

Supplementary Figure S4. Gene expression profiling of PDX tumors. **A-C**, Extended data associated with Fig. 4C,D. **A**, Box plots showing the number of genes with upregulated, downregulated, and unchanged expression in treatment group comparisons. **B,** Number of significantly downregulated genes shared between treatment groups. **C**, GO 'Biological Process' terms downregulated in palbociclib + fulvestrant (P+F) compared to vehicle (V) treated tumors (top left panel), in AZD4573 versus vehicle (top right panel), in palbociclib + fulvestrant + AZD4573 (P+F+A) versus vehicle (bottom left panel) and in P+F+A versus P+F treated tumors (bottom right panel). **D**, Extended data associated with Fig. 4F,G; Expression of *BRD4* and *CCNE1* in tumor treatment groups (*p* values estimated using's edgeR's implementation of quasi-likelihood F-test).
