# Supplementary material for: Targeting Transcriptional Regulation with a CDK9 Inhibitor Suppresses Growth of Endocrine- and Palbociclib-Resistant ER+ Breast Cancers
Source: Cancer Res. 2023 Oct 6;84(1):17–25. doi: 10.1158/0008-5472.CAN-23-0650 (PMC10758688; doi:10.1158/0008-5472.CAN-23-0650)
Supplement: Supplementary Figure S5 [file can-23-0650_supplementary_figure_s5_suppsf5.pdf]

Supplementary Figure 5

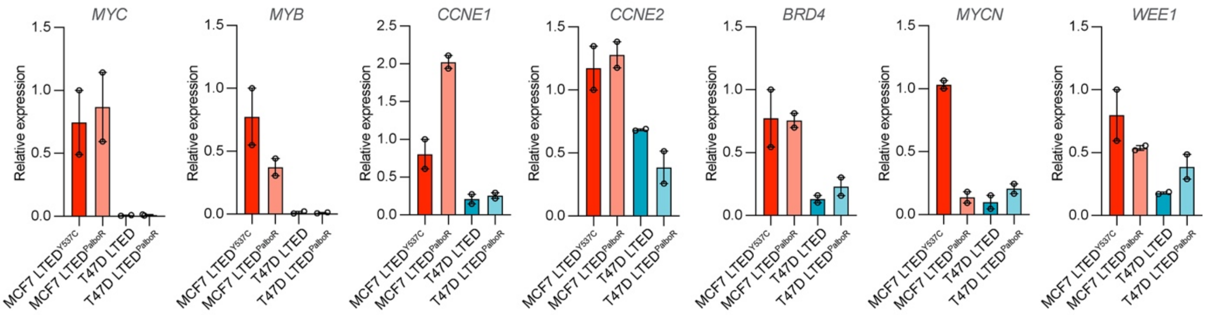

Supplementary Figure S5. RTqPCR analysis of cell lines. RTqPCR analysis of *MYC*, *MYB*, *CCNE1, CCNE2, BRD4, MYCN* and *WEE1* expression in MCF7 LTED and T47D LTED and their respective palbociclib resistant lines (n=2 biological replicates, n=3 technical replicates, ±SEM per cell line; two-tailed, unpaired t-test). Assays IDs: *B2M*, Hs00187842_m1; *BRD4*, Hs04188087_m1; *CCNE1*, Hs01026536_m1; *CCNE2*, Hs00180319_m1; *MCL1*, Hs00766187_m1; *MYB*, Hs00920556_m1; *MYC*, Hs00153408_m1; *MYCN*, Hs00232074_m1; *WEE1*, Hs00268721_m1.
